# PDK1 induces JunB, EMT, cell migration and invasion in human gallbladder cancer

**DOI:** 10.18632/oncotarget.4931

**Published:** 2015-08-05

**Authors:** Shixian Lian, Yebo Shao, Houbao Liu, Junyi He, Weiqi Lu, Yong Zhang, Ying Jiang, Jun Zhu

**Affiliations:** ^1^ Department of General Surgery, Zhongshan Hospital, Fudan University, Shanghai 200032, China

**Keywords:** 3-phosphoinositide-dependent protein kinase 1, JunB, invasion, migration, epithelial–mesenchymal transition

## Abstract

The protein 3-phosphoinositide-dependent protein kinase 1 (PDK1) is upregulated in cancer. Here we showed that PDK1 stimulated cell proliferation, invasion and metastasis in gallbladder cancer (GBC), by inducing JunB and epithelial–mesenchymal transition. JunB levels were increased in GBC samples and positively correlated with PDK1 levels in tumors. High levels of JunB predicted poor overall survival in GBC patients. Thus, PDK1 functions as a tumor promoter in human GBC by upregulating JunB.

## INTRODUCTION

Gallbladder cancer (GBC) is the most frequent cancer of the biliary tract and the most common neoplasm of the digestive system [1, 2]. Despite recent progress in diagnostic and therapeutic approaches, its 5-year overall survival (OS) is generally low [3]. The main reason for this is that relatively few patients with GBC are diagnosed prior to surgery [4, 5]. This late diagnosis leads to many GBC cases being unresectable at the time of presentation [6]. The development of new effective early tumor biomarkers would help detect GBC earlier and increase survival rates. The protein 3-phosphoinositide-dependent protein kinase 1 (PDK1) binds to PIP3 and facilitates the colocalization of Akt and PDK1 [7, 8]. Akt plays a key role in the regulation of various physiological processes related to metabolism, growth, proliferation, and survival. A variety of factors influence the cellular distribution of PDK1; for example, binding to soluble inositol phosphates causes PDK1 localization in the cytosol, where many of its non-phosphoinositide-binding substrates reside [9]. PDK1 has been detected in the nucleus as well [10].

PDK1 plays a crucial role in cell growth and metabolism. At the cellular level, PDK1 affects insulin-induced glycogen synthesis, membrane translocation of glucose transporter (GLUT) 4 and synthesis of related proteins, and cell survival [11]. Previous studies have shown that PDK1 may be a viable cancer biomarker [12]. Expression of PDK1 induces anchorage-independent growth *in vitro*, a hallmark of cellular transformation, and injecting PDK1 cell into nude mice induces homologous, poorly differentiated cells, leading to the formation of breast cancer [13]. PDK1-induced tumorigenesis may result from the inhibition of one or more of its downstream targets, such as Akt or PKC. The PDK1 substrates Akt1, Akt2, and Akt3 are highly expressed in several types of human cancers [14, 15]. Akt2 and PKC-α transform rat fibroblasts *in vitro*; however, no tumorigenesis has been observed [16, 17]. Despite these findings, a direct functional link between PDK1 activation and tumorigenesis has not yet been identified.

Another protein that may influence GBC invasion and metastasis is the transcription factor JunB. The JunB gene was originally discovered as an immediate early growth response gene in mammalian cells. Earlier studies suggested that JunB inhibits cell proliferation and migration by antagonizing c-Jun activity. JunB and c-Jun share extensive homology within the leucine zipper and basic domains, and JunB has been reported to rescue a lethal phenotype in c-Jun-null mice. c-Jun is a strong transcriptional activator, whereas JunB is a modest transactivator and may even repress transcription [18]. Both c-Jun and JunB can be induced in epithelial cells using TGF-β, whereas mesenchymal cells respond with the upregulation of JunB but not c-Jun [19]. JunB, together with TGF-β, induces epithelial-mesenchymal transition (EMT) and fibrotic responses in mammary and kidney epithelial cells [20].

In this study, we investigated the role of PDK1 in GBC, by evaluating changes in GBC cell morphology after both increasing and decreasing PDK1 expression via plasmid transfection and siRNA administration, respectively. We also examined the effect of PDK1 levels on JunB expression and on markers of EMT.

## RESULTS

### PDK1 is overexpressed in human GBC

**Figure 1:**
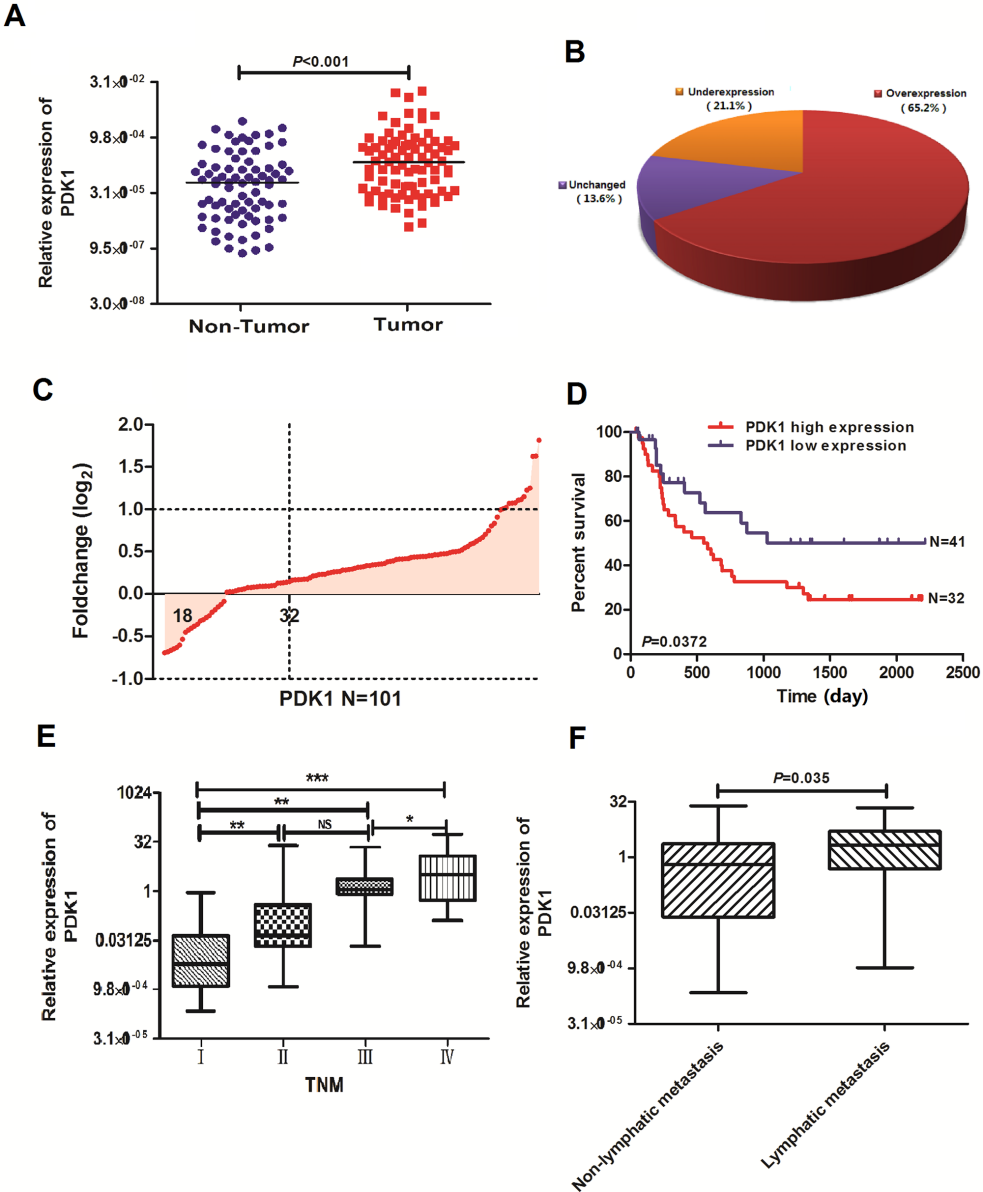
PDK1 expression is upregulated in GBC **A.** Protein 3-phosphoinositide-dependent protein kinase 1 (PDK1) expression was detected using quantitative real-time polymerase chain reaction (QRT-PCR) in 101 paired gallbladder cancer (GBC) and adjacent noncancerous tissues (NCTs). PDK1 expression was markedly upregulated in tumor tissues relative to the levels in the corresponding NCTs. **B.** PDK1 expression was upregulated in 65.2%, downregulated in 21.1%, and unchanged in 13.6% of the GBC samples. **C.** Upregulation of PDK1 in 101 primary GBC relative to the levels in the paired NCTs. **D.** Overall survival analysis based on the expression level of PDK1 in GBC tissues. **E.** Overexpression of PDK1 in primary GBCs of advanced stage relative to the levels in GBCs of lower stage. *p < 0.05, **p < 0.01, ***p < 0.001 **F.** Overexpression of PDK1 in primary GBCs with lymphatic metastasis relative to the levels in GBCs with nonlymphatic metastasis.

PDK1 levels were measured using quantitative real-time polymerase chain reaction (QRT-PCR) in an expanded cohort of 101 GBC patients. PDK1 was overexpressed in 66 (65.2%) of 101 GBC tissues compared to XX% in the corresponding noncancerous tissues (NCTs) (*p* < 0.001, Figures 1A and 1B). Moreover, PDK1 tumor-to-normal expression ratios were associated with the OS of GBC patients (*p* < 0.05, Figure 1D). In addition, PDK1 expression was associated with TNM pathological stage (*p* < 0.005, Figure 1E). PDK1 expression correlated with lymphatic metastasis. There was no significant association between PDK1 expression in GBC and sex, age, differentiation grade, lymph node status, or distant metastasis (Table 1).

**Table 1:** Relationship between PDK1 expression and clinicopathologic factors of patients with gallbladder cancer

Parameter	No. of patients	PDK1(low)	PDK1 (high)	*P* -value
Sex				0.2048
male	76	29	45	
female	25	9	16	
Age (yr)				0.1760
<60	68	25	33	
≥60	33	12	21	
Tumor differentiation				**0.0093**
I	6	1	5	
II	65	18	47	
III	30	13	17	
Tumor size (cm)				0.0513
≤5	61	29	32	
>5	59	31	28	
Differentiation grade				0.41254
Well-moderate	54	23	31	
Poor-undifferentiation	47	19	28	
T stage				**0.03641**
T1-T3	85	26	59	
T4	16	5	11	
Lymph node status				
Negative	56	33	22	0.18390
Positive	45	16	35	
Distant metastasis				0.2137
M0	59	33	26	
M1	42	24	18	
TNM stage				**0.0347**
I-II	37	17	20	
III-IV	64	21	43	
Lymphatic invasion				**0.035**
Negative	32	13	19	
Positive	69	24	45	
Venous invasion				**0.0351**
Negative	49	28	21	
Positive	52	35	17	

*P < 0.05

### PDK1 promotes cell invasion and metastasis both *in vitro* and *in vivo*

A consistently high expression of PDK1 in GBC suggests that it contributes to tumorigenesis. In a cell function experiment, we measured the expression of PDK1 in 5 different GBC cell lines using Western blot; bot high and low PDK1 expression was found in one cell line each (Supplementary Figure 1A). Subsequently, we constructed a PDK1 overexpression plasmid and siRNA-PDK1 for further studies. Western blot showed that the siRNA-PDK1 successfully decreased PDK1 protein levels (Supplementary Figure 1A); furthermore, after transfection with an overexpression plasmid, the protein levels of PDK1 significantly increased in comparison with the vector plasmid (i.e., the negative control). An *in vitro* proliferation assay revealed that the overexpression of PDK1 increased the growth rate of GBC-SD (*p* < 0.05, Supplementary Figure 1B), and a colony formation assay confirmed that PDK1 overexpression promoted the proliferation of GBC-SD cells (*p* < 0.02, Supplementary Figure 1B). In contrast, the silencing of PDK1 expression by siRNA reduced the growth of NOZ cells (*p* < 0.05, Supplementary Figure 1B).

**Figure 2:**
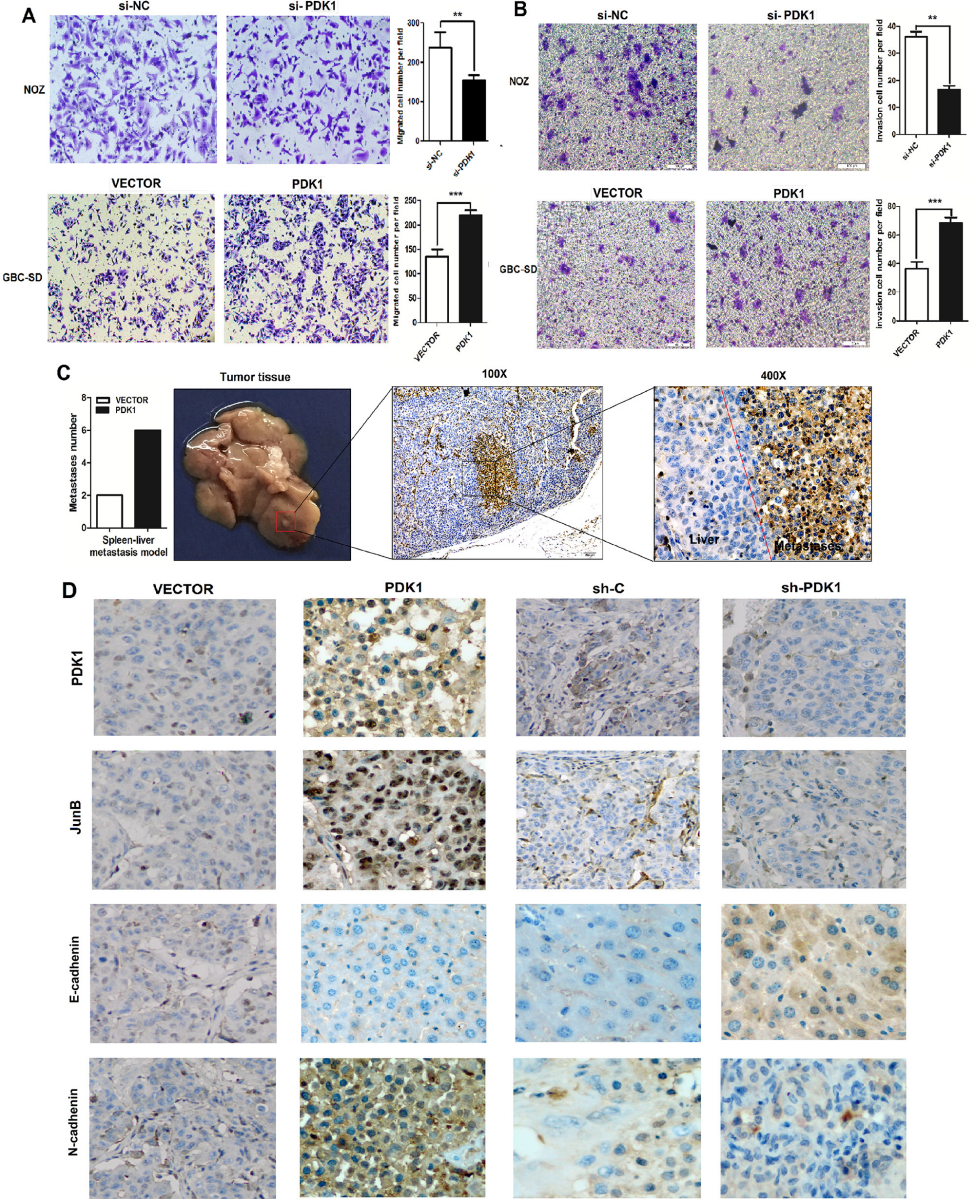
PDK1 promotes GBC cell invasion and metastasis both *in vitro* and *in vivo* **A.** siRNA knockdown of PDK1 in NOZ decreased cell metastasis, whereas overexpression of PDK1 enhanced metastasis in GBC-SD cells. *p < 0.05, **p < 0.01 **B.** siRNA knockdown of PDK1 in NOZ decreased cell invasion, whereas overexpression of PDK1 enhanced invasion of GBC-SD cells. *p < 0.05, **p < 0.01 **C.** The liver metastasis tumor model via spleen injection was employed to evaluate the pro-metastatic role of PDK1 (n = 10 for each group). The total metastasis number of each group is shown in histograms. Right panel: CA19–9 assay of liver metastatic nodule via IHC staining (400 ×). *p < 0.05, **p < 0.01 **D.** Paraffin sections stained with hematoxylin and eosin or used for IHC staining with anti-PDK1, Jun B, E-cadherin, and N-cadherin antibodies.

To determine if PDK1 could modulate the metastatic ability of GBC, we examined the effect of PDK1 on CRC cell invasion using a transwell assay. As shown in Figures 2A and 2B, PDK1-transfected CRC cells exhibited faster invasion and metastasis than the control cells, whereas the silencing of PDK1 suppressed the invasion and metastasis of NOZ cells. We then employed a liver tumor metastasis model using spleen injection of stable GDC-SD cell lines transfected with PDK1 to monitor their metastatic ability *in vivo*. About 2,500,000 GBC-SD cells were injected into nude mice. Three weeks after inoculation, the total metastasis occurrence number was much higher in the PDK1 overexpression group compared to the vector group (Figure 2C). After injection, we analyzed the dorsal subcutaneous tissues of nude mice that showed stable overexpression or knockdown of PDK1 in GBC-SD cells. Using immunohistochemical (IHC) staining, we found membrane localization of E-cadherin, N-cadherin, Jun B, and PDK1 in mice injected with the control plasmids (Figure 2D). In the PDK1 overexpression group, levels of N-cadherin increased and levels of E-cadherin decreased, compared to the control group. The opposite result was observed in the PDK1 knockdown group. These results suggest that PDK1 is closely associated with EMT markers and that PDK1 upregulation can accelerate GBC cell invasion and metastasis.

### PDK1 promotes cell invasion and metastasis through JunB regulation of EMT

**Figure 3:**
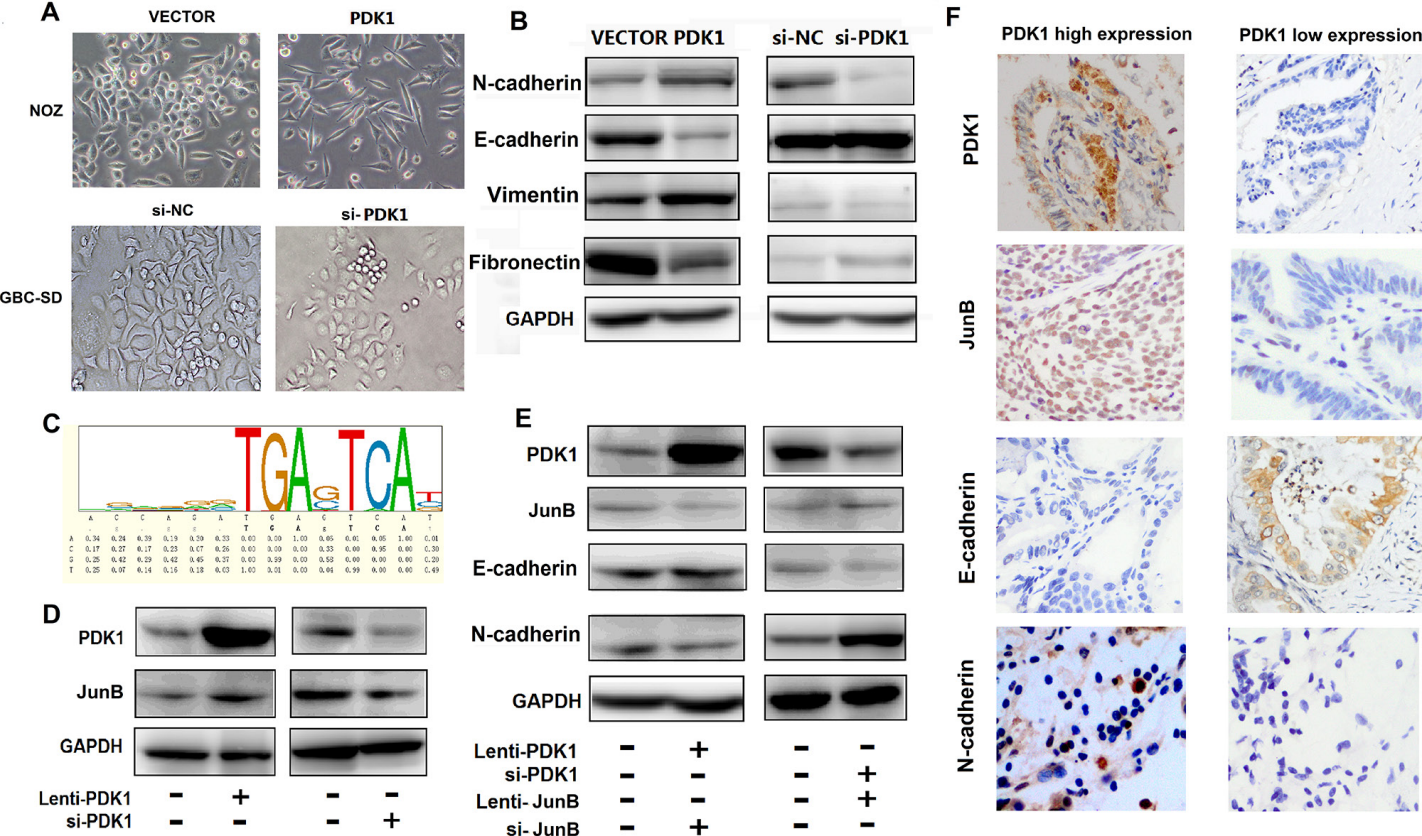
PDK1 induces JunB, which increases EMT in GBC **A.** NOZ cells transfected with PDK1 overexpression plasmid and cells transfected with vector plasmid are spindle shaped; si-PDK1 transfection caused the cells to become round. **B.** The protein levels of E-cadherin, FN1, vimentin and N-cadherin were compared between cells transfected with PDK1 and empty vector. The protein levels of E-cadherin, FN1, vimentin and N-cadherin were compared between cells transfected with si-PDK1 and si-NC. **C.** JunB binds to the DNA consensus sequence ACCAGATGAGTCAT at PDK1 downstream. **D.** JunB was upregulated when PDK1 was over expressed; it was downregulated following siRNA knockdown of PDK1. **E.** The protein levels of E-cadherin and N-cadherin did not change after combined transfection with PDK1 overexpression vector and si-JunB; however, the protein levels of E-cadherin and N-cadherin did after combined transfection with si-PDK1 and JunB overexpression vector. **F.** Representative PDK1, N-cadherin, E-cadherin, and Jun B expression in human GBC tissues with high or low PDK1 levels (400 ×).

NOZ cells transfected with either the PDK1 overexpression plasmid or the plasmid control remained spindle-shaped; however, cells transfected with si-PDK1 became round in shape (Figure 3A). This suggests that PDK1 may have an effect on EMT. Therefore, we quantified the expression of EMT markers. We used E-cadherin and fibronectin 1 (FN1) as epithelial cell phenotype markers and N-cadherin and V-catenin as mesenchymal cell phenotype markers. Figure 3B shows that E-cadherin and FN1 expression decreased, whereas N-cadherin and V-catenin expression increased, following PDK1 overexpression. To investigate the molecular mechanism by which PDK1 accelerates GBC cell invasion and metastasis, we used the UCSC database (http://genome.ucsc.edu) to determine its interaction with transcription factors. We found that transcription factor JunB binds to the DNA consensus sequences ACCAGATGAGTCAT at chr2:173417684–173417697 downstream of the PDK1 gene (Figure 3C). To further evaluate whether JunB is a direct functional target of PDK1 in GBC, we tested JunB expression after transfection with the PDK1 overexpression plasmid and showed that it was upregulated. Moreover, JunB was downregulated when the expression of PDK1 was inhibited by siRNA (Figure 3D). Furthermore, transfection of a JunB overexpression plasmid in GBC cells with siRNA-mediated PDK1 knockdown restored the decrease in E-cadherin and increase in N-cadherin seen following PDK1 overexpression (Figure 3E). IHC staining using anti-PDK1, N-cadherin, JunB, and E-cadherin antibodies in human GBC tumor tissue with high or low PDK1 levels revealed expression patterns similar to those found in cell line and animal model experiments (Figure 3F). Taken together, these data suggest that PDK1 promoted cell invasion and metastasis in GBC through the upregulation of JunB and promotion of EMT.

### PDK1 levels positively correlated with JunB expression and poor prognosis in GBC

**Figure 4:**
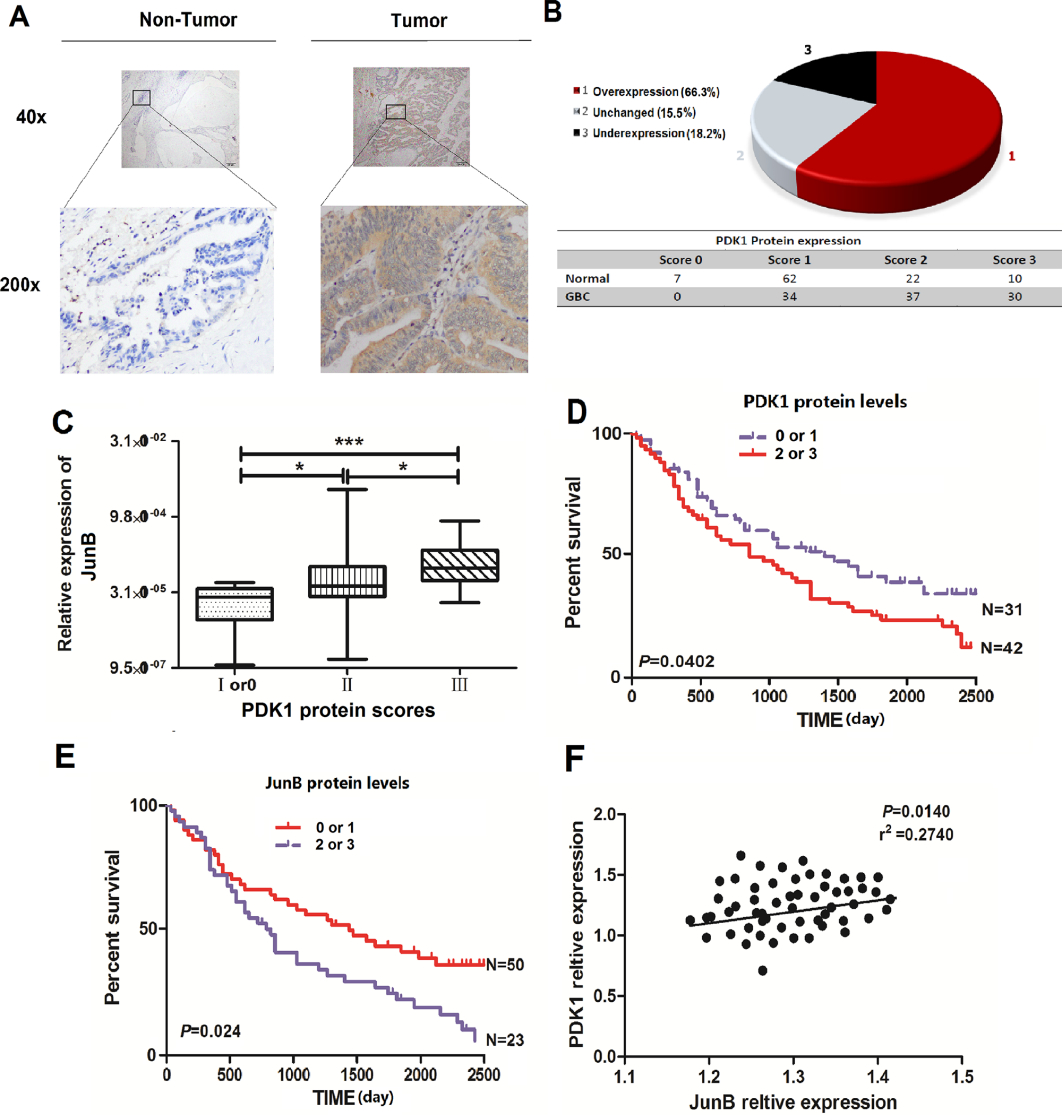
JunB is overexpressed in GBC and its levels are positively correlated with levels of PDK1 **A.** Immunohistochemical staining of PDK1 in 101 GBC tumor tissues and the adjacent NCTs. Brown cytoplasmic PDK1 staining was observed in GBC tissues but was nearly absent in the normal epithelia. (original magnification, 40× or 200 ×) **B.** PDK1 is frequently upregulated in tumor tissues (*n* = 101) relative to the expression in matched NCTs, with overexpression in 66.3%, reduced expression in 15.5%, and unchanged expression in 18.2% of the GBC tissues. **C.** Levels of JunB positively correlated with PDK1 levels in GBC tissues. *p < 0.05, **p < 0.01 **D.** Overall survival analysis based on the expression level of PDK1. The groups were ranked according to the PDK1 staining intensity. Patients scored as 0 or 1 were included in the low-expression group, whereas those who scored 2 or 3 were included in the high-expression group. The overall survival was significantly higher in patients with low PDK1 expression (score of 0 or 1) than in patients with high PDK1 expression (scores of 2 or 3). **E.** Overall survival analysis based on the expression level of JunB. The groups were ranked according to the JunB staining intensity. Patients who scored 0 or 1 were included in the low-expression group, whereas those who scored 2 or 3 were included in the high-expression group. The overall survival was significantly higher in patients with low JunB expression (score of 0 or 1) than in patients with high JunB expression (score of 2 or 3). **F.** Levels of JunB positively correlated with the PDK1 levels in GBC tissues (p = 0.0140, r^2^ = 0.2740).

To study the relationship between PDK1 and JunB in human GBC, we quantified levels of PDK1 in the 101 paired human GBC and NCT samples using IHC. Compared to the matched NCTs, PDK1 levels were increased in GBC tissue and were positively correlated with JunB levels (Figure 4A). PDK1 was overexpressed in 66.3% (67/101) of GBC tissues compared to XX% of matched NCTs (*p* < 0.05, Figure 4B), and protein expression was significantly associated with TNM pathological stage (*p* < 0.05, Figure 4C). More importantly, enhanced immunoreactivity of PDK1 and JunB in GBC was inversely correlated with OS and suggested a poor prognosis for CRC patients (*p* < 0.05, Figures 4D and 4E). PDK1 expression was positively correlated with JunB levels (*p* < 0.05, Figure 4F).

## DISCUSSION

PDK1 plays a key role in the signaling pathways activated by several growth factors and hormones and activates members of the AGC family of protein kinases [21–23]. Changes in the expression and activity of PDK1 and several AGC kinases have been linked to human diseases, including cancer [12, 24, 25]. A recent study found that the upregulation of PDK1 promotes breast cancer oncogenesis, increases breast cancer progression and cell migration, and promotes experimental metastasis [26, 27]. Moreover, PDK1 has been suggested as a viable target in head and neck cancer, multiple myeloma, pancreatic cancer, and colorectal cancer [28–32]. However, the exact function of PDK1 in GBC is largely unknown. Our results indicate that PDK1 is upregulated in GBC. The clinical data of 101 patients with GBC were retrospectively analyzed, and we found that the overexpression of PDK1 was associated with tumor differentiation, T stage, TNM stage, lymphatic invasion, and venous invasion (*p* < 0.05). However, PDK1 overexpression was not significantly associated with tumor size, differentiation grade, lymph node status, age, or sex (Table 1). Univariate and multivariate analyses showed that PDK1 overexpression was significantly associated with differentiation grade, T stage, lymph node status, TNM stage, and lymphatic invasion (*p* < 0.05), but was not significantly associated with sex, age, tumor size, distant metastasis, or venous invasion (Tables 2 and 3). On the basis of these results, we suggest upregulation of PDK1 could be an important carcinogenic factor in GBC. In addition, the results indicate that upregulation of PDK1 is associated with GBC invasion and metastasis. Therefore, we speculate that PDK1 could be an important prognostic marker for GBC. Functional studies have provided the first line of evidence that PDK1 can significantly promote GBC invasion and metastasis. However, we found that PDK1 was not significantly associated with the proliferation of GBC. Reports have stated that PDK1 can promote cancer cell proliferation through PDK1-Akt/PKB-TSC2-MTORC1 signaling [33].

**Table 2:** Univariate analysis identifies factors influencing the overall survival rate of gallbladder cancer patients

Factors	HR	95% CT	P value
Sex	0.897	1.05–2.57	0.389
Age (> 60 vs. ≤ 60)	1.462	0.701–2.967	0.893
Tumor size(cm) > 5	5.813	5.024–10.54	0.0324
Tumor size(cm) < 5	3.124	1.234–3.002	0.0627
Differentiation grade	2.341	1.689–3.762	**0.0201**
T stage	1.982	1.152–2.641	**0.0031**
Lymph node status	3.124	1.10–2.98	**0.046**
Distant metastasis	2.58	0.789–2.315	0.452
TNM stage (IIIvs.II vs.I)	1.512	1.025–3.112	**0.0103**
Lymphatic invasion	1.67	1.07–3.024	**0.0071**
Venous invasion	1.87	1.10–3.21	0.391
PDK1 expression	2.76	1.85–2.881	**0.0017**

HR: hazard ratio; CI: confidence interval; TNM: tumor–node–metastasis classifications.

**Table 3:** Multivariate analysis identifies factors influencing the overall survival rate of gallbladder cancer patients

Factors	HR	95% CT	*P* value
Tumor size(cm) > 5 vs. ≤ 5	1.96	1.31–2.89	0.057
Differentiation grade	2.13	1.02–2.57	0.320
T stage	0.89	0.64–2.25	**0.01**
Lymph node status	2.75	1.24–3.54	**0.024**
TNMstage(IIIvs.II vs.I)	1.35	1.20–1.88	**0.004**
Lymphatic invasion	0.99	1.10–3.20	**0.001**
Venous invasion	1.012	2.31–3.501	0.389
PDK1 expression	2.33	1.25–3.61	**0.001**

HR: hazard ratio; CI: confidence interval; TNM: tumor–node–metastasis classifications.

While investigating the mechanism underlying the promotion of GBC invasion and metastasis by PDK1, we found that GBC cells underwent morphological changes, and we detected increases in EMT marker levels; therefore, we investigated the downstream regulation transcription factors. We found evidence that the JunB transcription factor, the expression of which is affected by PDK1 levels, is a key regulator of GBC. Furthermore, we showed that JunB protein expression decreased following the inhibition of PDK1 expression by siRNA. Moreover, we found that overexpression of PDK1 did not alter N-cadherin and E-cadherin levels when JunB expression was inhibited by siRNA. These results were confirmed with an *in vivo* assay. In addition, we used IHC analysis to show that PDK1 was coexpressed with JunB. Therefore, we propose that PDK1 regulates EMT by increasing JunB activity, ultimately promoting GBC invasion and metastasis. The authors of a previous study speculated that JunB may be regulated through TGF-β-Smad signaling [20]. The relationships between PDK1, JunB, and TGF-β-Smad expression and their effects on GBC will need to be clarified in future studies.

In summary, we determined that PDK1 is frequently upregulated in GBC and is responsible, at least in part, for GBC invasion and metastasis. JunB activity, which is increased following PDK1 upregulation, promotes EMT and likely plays a role in the observed effects of PDK1 overexpression. Based on our findings that PDK1 expression is closely related to the prognosis of GBC, additional studies of PDK1 and JunB as cancer biomarkers are warranted.

## MATERIALS AND METHODS

### Human tissues and cell lines

A total of 101 pairs of human primary GBC and their adjacent NCTs were collected between 2006 and 2012 at the Fudan University Zhongshan Hospital. The tissue samples were immediately snap-frozen in liquid nitrogen and histologically examined in a timely manner. All the human materials were collected after obtaining informed consent, and this study was approved by the Clinical Research Ethics Committee of the Fudan University Zhongshan Hospital. The clinical information of the GBC patients is presented in Table 1. The HEK-293T cell line and 5 human GBC cell lines, including GBC-SD, NT, SGC-996, NOZ, and OCUG, were purchased from the American Type Culture Collection (ATCC). All the media (Invitrogen, USA) were supplemented with 10% fetal bovine serum (Gibco, USA). The cells were incubated under the conditions recommended by ATCC.

### DNA and RNA extraction and QRT-PCR

Tissue genomic DNA was isolated using the DNeasy Blood and Tissue kit (Qiagen, German) according to the manufacturer's protocol. Total RNA was extracted using the TRIzol reagent (Invitrogen, USA) according to the manufacturer's instructions. The concentrations of RNA were determined in the samples using a NanoDrop ND-1000 instrument (NanoDrop, USA), and aliquots of the samples were stored at − 80°C. cDNA was synthesized using the PrimeScript RT reagent kit (TaKaRa, Japan) and 500 ng of total RNA as a template. QRT-PCR analyses were performed to quantitate mRNA relative expression using SYBR Premix Ex Taq (TaKaRa, Japan) with beta-actin as an internal control. The results of QRT-PCR were defined using the threshold cycle (Ct), and relative expression levels were calculated using the 2-△△Ct method. QRT-PCR was performed using an ABI 7900HT instrument (Applied Biosystems, USA). The primers used for QRT-PCR analysis are listed in the Supplementary Data (Table S1).

### Vector constructs

The open reading frame of the PDK1 and JunB sequences were amplified from normal human genomic DNA using nested PCR and PrimerSTAR Premix (TaKaRa, Japan). The sequence was then cloned into the lentivirus expression vector pWPXL (ThermoFisher, USA) to generate pWPXL-PDK1 and pWPXL-JunB. The primers and endonuclease sites used for the vector constructs are shown in the Supplementary Data (Table S1).

### Lentivirus production and transduction

Virus particles were harvested 48 h after cotransfecting pWPXL-PDK1 and pWPXL-JunB with the packaging plasmid ps-PAX2 and the envelope plasmid pMD2G into HEK-293T cells using Lipofectamine 2000 reagent (Invitrogen, USA). GBC-SD and NOZ cells were infected with recombinant lentivirus-transducing units plus 6 μg/mL polybrene (Sigma, USA).

### Oligonucleotide transfection

PDK1 and JunB small-interfering RNA (siRNA, target sequence: CAATGGCCCAGGGTGTGATTGAATA;TGGAGGACAAGGTGAAGACGCTCAA) were synthesized (Jinweizhi, China). Oligonucleotide transfection was performed using RNAiMAX reagents (Invitrogen, USA) according to the manufacturer's instructions. The final concentration of the siRNA in the transfection mixture was 50 nM.

### Cell proliferation and colony formation assays

Cell proliferation was quantified using a Cell Counting Kit-8 (CCK8; Dojindo Laboratories, Japan) according to the manufacturer's instructions. For the colony formation assays, 1500 cells/well of GBC-SD cells and 1500 cells/well of NOZ cells were plated into 6-well plates and incubated in a medium containing 10% fetal bovine serum for 10 days. The colonies were fixed with methanol and stained with 0.1% crystal violet in 20% methanol for 30 min. The number of colonies containing more than 30 cells was counted using an inverted microscope.

### Cell migration assay

For the migration assays, 2 × 10^5^ GBC-SD cells or 4 × 10^4^ NOZ cells (stably expressed pWPXL-PDK1 or vector control) in 200 μL serum-free medium were placed into the top chamber of each insert (BD Biosciences, NJ). After 24 (GBC-SD) or 36 (NOZ) h of incubation at 37°C, cells adhering to the lower membrane were stained using 0.1% crystal violet in 20% methanol, imaged, and counted using an IX71 inverted microscope (Olympus, Japan).

### Western blot and immunohistochemical (IHC) analysis

The cells were harvested, and proteins were first separated using 10% sodium dodecyl sulfate polyacrylamide gel electrophoresis and then transferred to nitrocellulose membranes (Bio-Rad Laboratories, USA). The membranes were blocked using 5% nonfat milk and incubated with mouse anti-PDK1 and anti-JunB polyclonal antibodies with a dilution of 1:1000 (CST, USA) or a mouse anti-glyceraldehyde-3-phosphate dehydrogenase (GAPDH) monoclonal antibody with a dilution of 1:1000 (Sigma, USA). The membranes were subsequently incubated using a goat anti-mouse horseradish peroxidase secondary antibody (Sigma, USA). The protein complex was detected using enhanced chemiluminescence reagents (Pierce, France). Endogenous GAPDH was used as the internal control. IHC analysis was performed using PDK1 (Santa Cruz, USA), E-cadherin (Abcam, USA), N-cadherin (Abcam, USA), and Jun B (Abcam, USA) antibodies and previously described methods [34]. Paraffin-embedded samples of GBC were constructed using 101 paired CRC tissues and NCTs. Immunohistochemical staining was performed on 4-μm sections of paraffin-embedded tissues to determine the expression level of ARL5A protein. In brief, the slides were incubated in PDK1 and JunB antibody (CST, USA) and diluted 1:500 at 4°C overnight. The subsequent steps were performed using the EnVision™ FLEX High pH visualization system according to the manufacturer's instructions.

### Xenograft tumor model in nude mice

The stable transfected cell line GBC-SD (used in the overexpression group and knockdown group) and the negative control GBC-SD vector were used to perform the experiments in both the subcutaneous xenograft and the liver tumor metastasis models that used spleen injection. In the subcutaneous xenograft tumor model, viable cells (2.5 × 10^6^ in 100 mL of phosphate buffered saline [PBS]) were injected subcutaneously into 4 groups (10 mice/group) of 6-week-old nude mice. Four weeks after tumor cell inoculation, the mice were sacrificed and the xenografted tumors were excised and examined by IHC analysis. In the liver tumor metastasis model using spleen injection, the GBC-SD cells were treated as previously described, and 1.0 × 10^6^ cells in 200 mL of PBS were then injected into the mouse livers via the spleen. After 5 weeks of incubation, the mice (10 mice/group) were sacrificed and their livers were harvested. Then, serial sections of liver specimens were subjected to hematoxylin and eosin staining to confirm the metastases. The metastases were counted.

### Statistical analyses

The results are presented as the mean values ± SEM. The data were subjected to Student's *t*-tests unless otherwise specified (χ2 test, Pearson's correlation). The overall survival curves were plotted according to the Kaplan–Meier method, with the log-rank test applied for comparisons. A *p* value of < 0.05 was considered to indicate statistical significance. SPSS 16.0 package (IBM, USA) and Graphpad prism 5.0 software (GraphPad Software, USA) were used for statistical analyses and scientific graphing, respectively.

## SUPPLEMENTARY FIGURES AND TABLES


