# Supplementary material for: PDK1 induces JunB, EMT, cell migration and invasion in human gallbladder cancer
Source: Oncotarget. 2015 Aug 5;6(30):29076–86. doi: 10.18632/oncotarget.4931 (PMC4745712; doi:10.18632/oncotarget.4931)
Supplement: Supplementary file 1 [file oncotarget-06-29076-s001.pdf]

SUPPLEMENTARY FIGURE AND TABLE

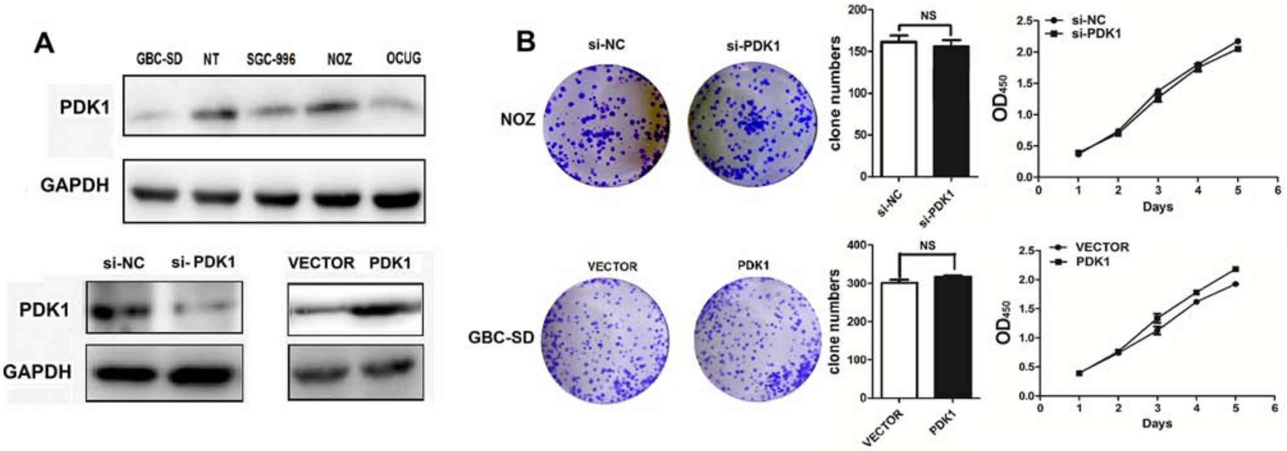

Supplementary Figure S1: A. Expression of PDK1 was detected in 5 GBC cell lines using Western blot; shown is the expression of eIF5A2 after siRNA knockdown relative to the expression of si-NC and the overexpression of pWPXL-PDK1 relative to that of the cells transfected with the vector. The protein levels of PDK1 were detected using Western blot. **B.** siRNA knockdown of PDK1 in NOZ cells decreased colony formation rates relative to those in the control cells. Stable expression of PDK1 in GBC-SD tended to increase colony formation rate relative to that of the control cells, but the differences were not significant; the cell counting Kit-8 assay was used to determine the cell growth rate and showed the same result. *$p < 0.05$, **$p < 0.01$, compared to the negative control (NC).

Supplementary Table S1: Sequences of primers and siRNA

**Real time PCR primers**	
PDK1-F	TGAAGATGAGTGACCGAGG
PDK2-R	GCAATCCATAACCAAAACC
JUNB-F	CTACTCCCCAGCCTCTGCGT
JUNB-R	GGTCTGCGGTTCCTCCTTG
**Sequences of siRNA**	
si-PDK1	CAATGGCCCAGGGTGTGATTGAATA
SI-JunB	GCCCAGCTCAAACAGAAGGTCATGA
